# Molecular basis of the selective processing of short mRNA substrates by the DcpS mRNA decapping enzyme

**DOI:** 10.1073/pnas.2009362117

**Published:** 2020-07-28

**Authors:** Anna-Lisa Fuchs, Jan Philip Wurm, Ancilla Neu, Remco Sprangers

**Affiliations:** ^a^Department of Biophysics I, Regensburg Center for Biochemistry, University of Regensburg, 93053 Regensburg, Germany;; ^b^Max Planck Institute for Developmental Biology, 72076 Tübingen, Germany

**Keywords:** enzyme regulation, mRNA decay, NMR spectroscopy, conformational changes, scavenger decapping enzyme

## Abstract

The 5′ messenger RNA (mRNA) cap structure enhances translation and protects the transcript against exonucleolytic degradation. During mRNA turnover, this cap is removed from the mRNA. This decapping step is catalyzed by the Scavenger Decapping Enzyme (DcpS), in case the mRNA has been exonucleolyticly shortened from the 3′ end by the exosome complex. Here, we show that DcpS only processes mRNA fragments that are shorter than three nucleotides in length. Based on a combination of methyl transverse relaxation optimized (TROSY) NMR spectroscopy and X-ray crystallography, we established that the DcpS substrate length-sensing mechanism is based on steric clashes between the enzyme and the third nucleotide of a capped mRNA. For longer mRNA substrates, these clashes prevent conformational changes in DcpS that are required for the formation of a catalytically competent active site. Point mutations that enlarge the space for the third nucleotide in the mRNA body enhance the activity of DcpS on longer mRNA species. We find that this mechanism to ensure that the enzyme is not active on translating long mRNAs is conserved from yeast to humans. Finally, we show that the products that the exosome releases after 3′ to 5′ degradation of the mRNA body are indeed short enough to be decapped by DcpS. Our data thus directly confirms the notion that mRNA products of the exosome are direct substrates for DcpS. In summary, we demonstrate a direct relationship between conformational changes and enzyme activity that is exploited to achieve substrate selectivity.

## Significance

In eukoryotes, 3′ to 5′ mRNA degradation is a major pathway to reduce mRNA levels and, thus, an important means to regulate gene expression. Herein, messenger RNA (mRNA) is hydrolyzed from the 3′ end by the exosome complex, producing short capped RNA fragments, which are decapped by DcpS. Our data show that DcpS is only active on mRNA that have undergone prior processing by the exosome. This DcpS selection mechanism is conserved from yeast to humans and is caused by the inability of the enzyme to undergo structural changes that are required for the formation of a catalytically active state around long mRNA transcripts. Our work thus reveals the mechanistic basis that ensures an efficient interplay between DcpS and the exosome.

**Fig. 1.**
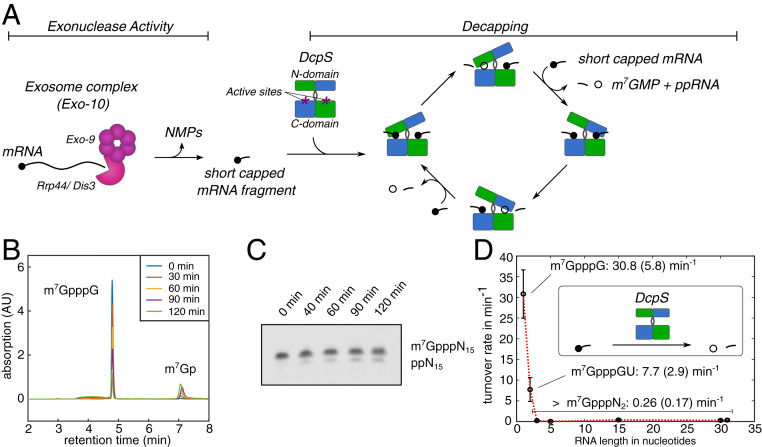
Substrate length dependency of DcpS turnover rates. (*A*) mRNA is hydrolyzed in the 3′ to 5′ direction by the exosome complex (pink) that releases short capped RNA fragments. The exosomal products are the substrates for DcpS (green/blue). The homodimeric DcpS enzyme has two active sites located between the N- and C-terminal domains (purple asterisks). Upon substrate interaction (short capped mRNA fragment), the enzyme undergoes a conformational change and forms one closed site that is catalytically competent. After catalysis in one active site, the N-terminal domain flips to close the second active site and release the products m^7^GMP (open circle) and the decapped RNA fragment. (*B*) HPLC profile that was used to determine DcpS activity for an mRNA substrate with an RNA body of 1 nucleotide (see also *SI Appendix*, Fig. S1). This approach was used for substrates that were between 1 and 5 bases long. (*C*) Exemplary polyacrylamide gel electrophoresis (UREA-PAGE) gel that is used to determine DcpS activity for mRNAs with an RNA body of 15 or more nucleotides (see also *SI Appendix*, Fig. S1). (*D*) The activity of DcpS strongly depends on the length of the mRNA body. The enzyme shows biologically relevant activity only for capped mRNAs with a body of one or two nucleotides. Longer mRNAs (3-, 5-, 15-, or 31-nucleotide body) are not efficiently processed by DcpS.

The accurate regulation of gene expression is essential in order to maintain cellular homeostasis. A final and irreversible way to terminate gene expression is the degradation of an mRNA transcript. An mRNA molecule is divided into the mRNA body that includes the coding region as well as the 3′ and 5′ UTRs, the 3′ poly(A)-tail, and the protecting 5′ cap structure. These elements are degraded in a sequential manner, where the removal of one feature triggers the next degradation step. For regular mRNA degradation the decay process is initiated with the gradual shortening of the 3′ poly(A)-tail by the Ccr4:Not and Pan2:Pan3 complexes (1–3). After this rate-limiting deadenylation step, the mRNA is rendered unstable and will be rapidly degraded in one of two complementary pathways. Both pathways comprise different enzymes and include an exonucleolytic step that degrades the mRNA body as well as a decapping step, that releases the 5′ cap structure from the mRNA. In the 5′ to 3′ pathway, the 5′ cap structure of the mRNA is first removed by the Dcp1:Dcp2 decapping complex (4–7), after which the mRNA body is degraded by the exoribonuclease Xrn1 (8). In the 3′ to 5′ pathway (Fig. 1*A*), the mRNA body is processively hydrolyzed by the 10-component cytoplasmic exosome complex (exo-10; exo-9 plus Rrp44/Dis3), followed by the removal of the cap structure of the remaining short RNA fragment by the Scavenger Decapping enzyme DcpS (Dcs1p in yeast) (9, 10). The Exo10 complex comprises the catalytically inactive Exo9 core and the Rrp44 enzyme (Dis3) that harbors both endonucleolytic and exonucleolytic activities. The exosome and DcpS have been shown to form a complex, and experiments in cell extracts suggest that DcpS is capable of decapping the products of the 3′ to 5′ degradation pathway (11). Whether the products are directly handed over from the exosome to DcpS or whether further enzymes are involved has not been addressed.

The apo DcpS decapping enzyme assembles into an 80-kDa symmetric homodimer that contains dimeric N- and C-terminal domains that are linked by a flexible hinge region (Fig. 1*A*) (10, 12, 13). The enzyme possesses two bipartite active sites that are located at the interface between the N- and C-terminal domains. Upon substrate binding to the C-terminal part of one of the active sites, the N-terminal domain flips over to enclose the mRNA substrate in a well-defined pocket. This motion simultaneously opens the other substrate binding site (10, 12, 13). Within the substrate loaded closed active site, hydrolysis of the bond between the alpha and beta phosphate of the triphosphate linkage is performed by the catalytic HIT motif, which produces m^7^GMP and diphosphorylated RNA (14–17). Subsequently, the enzyme undergoes a see-saw motion to simultaneously release the products from one binding site and capture a next substrate in the other active site (Fig. 1*A*).

On long mRNA substrates, DcpS should not be active, as this would interfere with the integrity of mRNA transcripts that are still actively translated. In agreement with that, it has been reported that the activity of DcpS is significantly and stepwise reduced if the length of the mRNA body increases from 1 to 10 nucleotides (17). This suggests that there is a length range for RNAs to be an eligible substrate for DcpS, which ensures that decapping of long RNA is prevented (16). Here, we determined the properties and the structural basis for such a substrate-length preference in DcpS, and we address how DcpS acts together with the exosome in the 3′ to 5′ degradation pathway.

In summary, we observe that DcpS is only able to efficiently digest capped mRNA substrates that have an RNA body of less than three nucleotides. Based on X-ray crystallography, methyl TROSY NMR spectroscopy (18–20), and biochemical assays, we find that this substrate length discrimination results from steric clashes between the third base in the mRNA body and parts of DcpS that prevent the formation of a closed and catalytically competent active site around longer mRNA substrates. This substrate-length selection mechanism is conserved from yeast to humans and is tuned such that the exosomal products are substrates for DcpS.

## Results

### DcpS Is Only Active on Very Short mRNA Substrates.

To determine the relationship between DcpS activity and substrate length, we made use of our recently developed method to prepare capped mRNA fragments of any length and sequence (21). Activity assays of DcpS were performed with capped RNA of a defined length, and turnover rates were determined (22). Based on these assays, we found that the activity of *Saccharomyces cerevisiae* DcpS on a capped RNA with a single nucleotide in the mRNA body is 30.8 ± 5.8 min^−1^ (Fig. 1 *B* and *D* and *SI Appendix*, Table S1 and Fig. S1), which is in agreement with previous reports (10). Increasing the length of the RNA body resulted in a decrease in the turnover rate: For a capped dinucleotide, the activity was reduced fourfold to 7.7 ± 2.9 min^−1^. For longer mRNAs (3-mer, 5-mer, 15-mer, 30-mer, and 31-mer) the decapping rate was reduced >100-fold to 0.26 (0.17) min^−1^ (Fig. 1 *C* and *D* and *SI Appendix*, Table S1 and Fig. S1). This shows that DcpS has an activity threshold between substrate lengths of 2 and 3 nucleotides (Fig. 1*D*). These results are partially in agreement with published data that describe a decrease in DcpS activity with increasing substrate length (17). Quantitatively, our data show, however, that the range in length for eligible substrates is much tighter than was previously suggested.

### Longer mRNAs Prevent the Formation of an Active DcpS Conformation.

The apo DcpS enzyme is a symmetric dimer in solution (Fig. 1*A*), which results in identical NMR chemical shifts for both protomers. Upon substrate binding, DcpS undergoes a conformational change (Fig. 1*A*) and a catalytically competent substrate bound state is formed (10). Such a conformational change can be directly observed in NMR spectra as the enzyme is then no longer in its symmetric conformation and the residues of both protomers give rise to different NMR resonances.

**Fig. 2.**
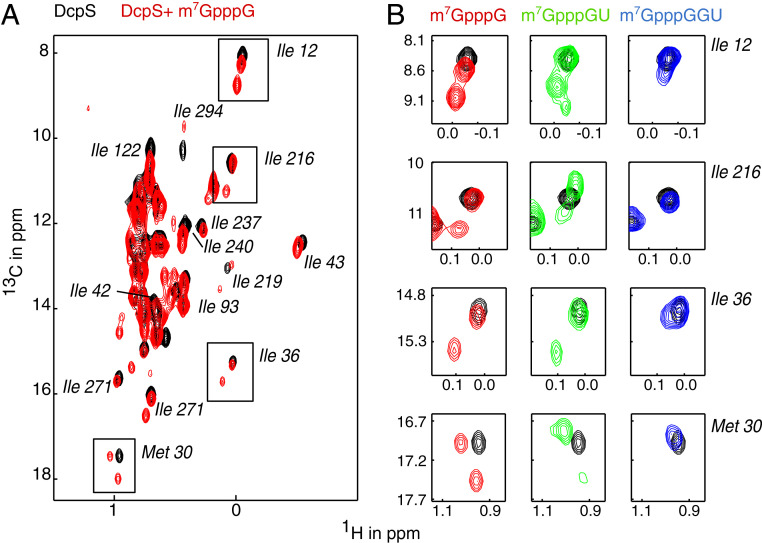
Substrates longer than two nucleotides do not induce a stable asymmetric conformation of DcpS. (*A*) Methyl TROSY NMR spectra of apo DcpS (inactivating H268N mutant) in the absence of substrate (black) and in the presence of m^7^GpppG (red). Peak splitting upon titration with m7GpppG indicates the conformational change from the symmetric apo enzyme to the asymmetric substrate-bound enzyme. Methyl group assignments are indicated. (*B*) Enlargement of the boxed resonances in *A* that report on the symmetric to asymmetric conformational change in DcpS. Titration with capped dinucleotide (green) induces the asymmetric conformation, while titration with capped trinucleotide (blue) retains the symmetric conformation.

As expected, we observe that DcpS undergoes a conformational change upon interaction with a capped mononucleotide (m^7^GpppG), which results in the formation of a catalytically competent state (10) (Fig. 2*A* and *SI Appendix*, Fig. S2*A*; note that a catalytically inactive H268N form of the enzyme is used to prevent hydrolysis of the cap structure; ref. 12). Likewise, we observe that DcpS adopts the catalytically active conformation upon interaction with a capped dinucleotide (m^7^GpppGU) (Fig. 2*B*, green). These data are in agreement with our activity assays (Fig. 1*D*), where we showed that the enzyme is active on these short substrates. On the contrary, DcpS does not form a stably closed active site upon interaction with a capped trinucleotide (m^7^GpppGGU) (Fig. 2*B*, blue). This observation is also in agreement with our activity assays (Fig. 1*D*) that show that DcpS is not able to efficiently remove the cap structure from this capped trimer. It is important to note that DcpS directly binds the capped trinucleotide, as we observe clear chemical shift perturbations (CSPs) in DcpS upon addition of m^7^GpppGGU. The lack of DcpS activity on this mRNA fragment is thus not due to the inability of the enzyme to interact with longer mRNAs (*SI Appendix*, Fig. S2*B*), but to the inability of DcpS to form a properly closed active site around longer mRNAs. In summary, we show that DcpS is only active on mRNA substrates that are compatible with the formation of a closed active site.

### The Third mRNA Nucleotide Prevents Formation of the Closed Active Site.

**Fig. 3.**
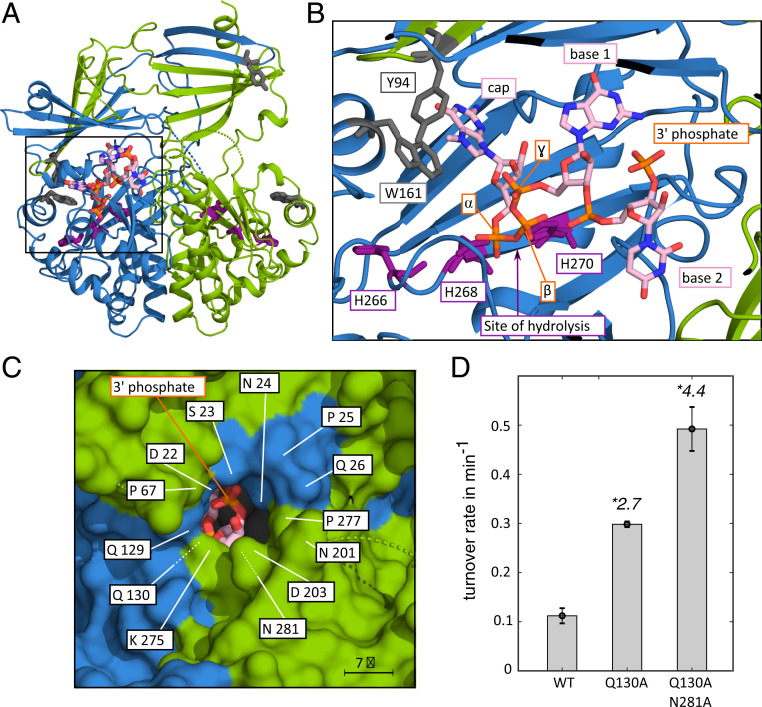
Structure of the DcpS:m^7^GpppGU complex. (*A*) Overview of the complex. The two DcpS protomers are colored blue and green (see also Fig. 1*A*). The RNA substrate (orange/pink) and the active site residues (purple) are shown as sticks. The protein adopts the asymmetric conformation, where the substrate is embedded in the catalytically competent active site (left side). The other side of the enzyme is open and in a catalytically incompetent state. (*B*) Enlargement of the boxed region in *A*. The methylated cap base interacts with W161 and Y94 (gray). H268, that is mutated to an N in our construct to inactivate the enzyme, is located close to the triphospate bond between the m^7^G cap and the mRNA body. The site of hydrolysis is indicated with an arrow. The first two bases of the mRNA are clearly defined and show the path that the RNA body takes toward the putative RNA exit hole. (*C*) View from the outside of the enzyme toward the active site. The 3′ phosphate group of the mRNA body is visible through the putative RNA exit hole and indicates the connection site for further RNA bases. The indicated residues in the exit tunnel restrict the space that would be required for a putative third base. (*D*) Mutations that enlarge the exit tunnel for the mRNA body increase the decapping activity of DcpS on long mRNA substrates. On long substrates the activity of the Q130A and Q130A N281A DPs enzymes are 2.7, respectively 4.4 times higher than the activity of the WT enzyme.

To obtain insights into the mechanism that prevents DcpS from forming a productive active site around mRNA substrates longer than two nucleotides, we determined the crystal structure of DcpS in complex with a capped dinucleotide (Fig. 3*A*; note: DcpS contains the H268N inactivating mutation). The complex crystallized in space group P2_1_2_1_2_1_, with two dimers per asymmetric unit. The structure was determined by molecular replacement (23) with the structure of yeast DcpS in complex with m^7^GDP (5BV3; ref. 10) as a search model. The final model was refined to 2.9-Å resolution with good geometry (*SI Appendix*, Table S2). In this structure, DcpS is in the asymmetric conformation, with the N-terminal lid domain flipped over such that the substrate is tightly embeded in the closed active site (Fig. 3*A*). In agreement with our activity assays, the catalytic triad is oriented such that the triphosphate linkage can be hydrolyzed. The methylated guanine cap structure is tightly embedded in the active site and stabilized by stacking interaction with Trp-161 (Fig. 3*B*). Importantly, the structure also displays clear density for the mRNA body of the capped dinucleotide (*SI Appendix*, Fig. S3). This reveals the path of the mRNA inside the closed active site and shows that the RNA body has no sequence specific interaction within the binding site. The second nucleotide points away from the cap structure, toward a narrow opening between the N- and C-terminal domains and fully occupies this region of the active site. The opening between the domains has previously been proposed as a possible RNA exit channel (12). Based on the path of the mRNA body, a third nucleotide would be placed inside this channel (Fig. 3*C*). However, with a diameter of ∼7 Å, the channel is too small to accommodate a putative third nucleotide. This is confirmed by modeling of a capped trinucleotide into the closed active site (*SI Appendix*, Fig. S4), which shows that the van der Waals radii of a third nucleotide and residues of the exit channel significantly overlap. From the DcpS side, these steric clashes are caused by sidechains in the loop opposing the channel and by sidechains and backbone atoms of residues at the outer part of the channel. Considerable conformational changes in the DcpS exit channel are thus required to accommodate a third nucleotide in the closed active site of DcpS. The energy that is associated with these conformational changes is likely too high to allow for stable formation of the active site and, thus, for effective catalysis of substrates longer than two nucleotides. This is in agreement with our NMR data that show that the stable asymmetric conformation in the enzyme is only formed with substrates up to two nucleotides in length.

The length of the DcpS exit channel is about the size of one nucleotide (*SI Appendix*, Fig. S4). This implies that nucleotides that follow the third nucleotide will not interfere with the DcpS closing mechanism. This finding is indeed reflected in our activity assays that show equal residual activity for mRNA substrates that have an RNA body of three nucleotides and those that are longer.

### Mutations in DcpS Can Alter the Substrate Length-Sensing Mechanism.

Next, we attempted to enlarge the mRNA exit channel through site-directed mutagenesis. We reasoned that an enlarged exit channel would be able to accommodate the third base in the mRNA. This would then make the closed and catalytically active conformation of the enzyme energetically more favorable and would thereby increase decapping activity on long mRNA substrates. To that end, we mutated Gln-130 and/or Asn-281 that are both located at the narrowest point of the pore (Fig. 3*C*), to Ala and performed decapping assays (Fig. 3*D* and *SI Appendix*, Fig. S5). Interestingly, the Q130A as well as the Q130A/N281A mutations result in an increased DcpS activity on a substrate that has an mRNA body of 15 nucleotides (2.7-fold and 4.4-fold, respectively, higher than the WT protein). Additional efforts to raise the activity even further failed, as the introduction of additional mutations reduced the stability of the enzyme and as main chain atoms also form a significant part of the narrow exit channel. Taken together, these results confirm that the size of the mRNA exit tunnel is central to the substrate selection mechanism of DcpS.

### The Length-Sensing Mechanism of DcpS Is Conserved among Species.

**Fig. 4.**
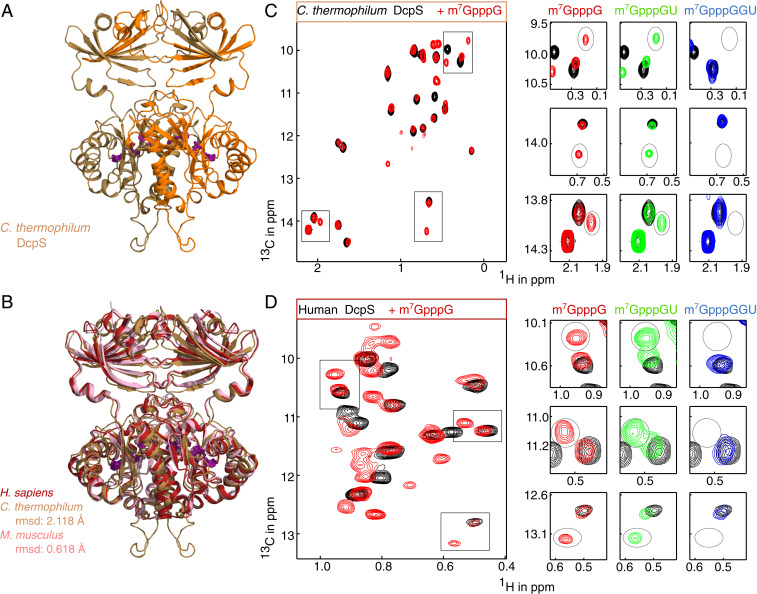
Structure and mechanism of DcpS from other organisms. (*A*) Structure of the apo-DcpS enzyme from *C. thermophilum*. The two chains are colored orange and brown. The catalytic triad is indicated in sticks (purple) and is located at the interface between the dimeric N- and C-terminal domains. The protein is in the apo conformation where both active sites are open. (*B*) Overlay of the apo structures of DcpS from human (13) (red; PDB ID code: 1XML), *C. thermophilum* (this work, brown; PDB ID code: 6GBS), and mouse (38) (pink; PDB ID code: 1VLR). The structures are highly similar, as indicated by the main-chain rmsd values. (*C*) Methyl TROSY NMR spectra of the *C. thermophilum* DcpS enzyme in the absence (black; symmetric) and in the presence of a capped monomer RNA (m^7^GpppG; red; asymmetric), a capped dimer RNA (m7GpppGU; green; asymmetric) or a capped trimer RNA (m7GpppGGU; blue; symmetric). The boxed regions in the *Left* spectrum are enlarged at *Right*, where the resonances of the catalytically compented closed state are marked with a dashed oval. (*D*) As in *C*, but for the human DcpS enzyme.

Above, we have focused on the DcpS enzyme from *S. cerevisiae* (Dcs1p) and identified the molecular basis by which the enzyme is able to selectively act on short mRNA substrates. Next, we aimed at determining whether this preference in substrate usage is a conserved mechanism, and we focused on the enzymes from C*haetomium thermophilum* (a thermophilic filamentous fungus) and humans. Structural data of the human enzyme in the apo and catalytically active state are available. Supplementarily, we here determined the structure of *C.t.* DcpS in the apo form (Fig. 4*A*). The protein crystallized in space group P1 with one dimer per asymmetric unit. The structure was determined by molecular replacement and refined to a resolution of 1.95 Å with good geometry (*SI Appendix*, Table S2). Apo *C.t.* DcpS forms a symmetric dimer with the same overall fold as the human and mouse protein (Fig. 4*B*), indicating that the fold and domain orientation of the protein is conserved despite a relatively low sequence conservation (*SI Appendix*, Fig. S6).

To test whether the *C. thermophilum* and human proteins also undergo the same conformational change as *S. cerevisiae* DcpS, we recorded methyl TROSY NMR spectra of these enzymes in the absence and presence of substrate of different length (Fig. 4 *C* and *D*). The *C. thermophilum* and human apo proteins show a single set of peaks (black contours), which confirms the findings from static crystal structures (Fig. 4 *B*–*D*). Upon addition of an mRNA substrate with one nucleotide (red contours), the *C. thermophilum* (Fig. 4*C*) and human (Fig. 4*D*) enzymes undergo a transition into an asymmetric conformation, thereby forming a catalytically competent active site. Likewise, an mRNA substrate with two nucleotides (green contours) induces a catalytically active conformation in the enzyme. On the contrary, an mRNA substrate of three nucleotides does not result in the formation of the closed conformation (Fig. 4 *C* and *D*; blue contours). From these data, we conclude that the length sensing mechanism that we identified in the *S. cerevisiae* protein is conserved in the *C. thermophilum* and human enzymes. This indicates that the mechanism determining the preference in substrate usage by DcpS is generally conserved among species.

### DcpS Decaps Short RNA Fragments Produced by the Exosome Complex.

During exosome-mediated mRNA decay, interactions between the long substrate and the exosome are lost. Eventually this will result in the release of short mRNA fragments (24). The length of these fragments is not precisely known but estimated to be roughly 4–5 nucleotides in length (24–27). According to our activity data (Fig. 1*D*), this length of a capped RNA cannot be processed at biologically relevant rates by DcpS. To test whether the products of exosomal degradation are, as is generally assumed (28), substrates for decapping by DcpS, we performed in vitro degradation assays. To that end, the recombinantly expressed and purified cytoplasmic exosome complex (exo-10) was mixed with capped RNA and the products were analyzed by ion exchange chromatography (*SI Appendix*, Fig. S7). This shows that the GA-rich capped RNA of 30 nucleotides is degraded into the nucleotides AMP, GMP, and a number of short mRNA products. Upon addition of DcpS, these mRNA products are further processed and the decapping product m^7^GMP is formed. This clearly shows that the exosome products can be further processed by DcpS and that these thus are shorter than 3 nucleotides. It is important to note that the DcpS-mediated decapping reaction only takes place after the mRNA is hydrolyzed by the exosome complex as the mRNA is not processed by DcpS in the absence of the exosome complex. From these data, we conclude that the cytoplasmic exosome produces substrates for the DcpS decapping enzyme and that a direct transfer of the mRNA fragments from the exosome to DcpS is possible.

## Discussion

In many biological pathways, a set of enzymes acts successively, where the product of one enzymatic reaction is the substrate for the next enzyme. This scenario also applies to mRNA degradation, which takes place in a regulated manner: The removal of the mRNA 5′ cap structure by DcpS only takes place after the exosome has degraded the mRNA body in the 3′ to 5′ direction. Here, we have addressed the mechanism, by which DcpS discriminates the length of potential substrates and whether exosome products are accepted as substrates by DcpS.

We show that the activity of DcpS depends strongly on the length of the substrate: Capped substrates with an mRNA body of 1 or 2 nucleotides are decapped with biologically relevant rates, whereas decapping rates for longer substrates are extremely low. In our assays, the drop in activity between the capped monomer and the capped dimer is about fourfold. In that light, it should be noted that our in vitro prepared capped dimer contains a 3′ phosphate group that results from the RNase A cleavage of the precursor RNA. In an in vivo setting, the DcpS substrates are produced by the exosome complex and will carry a 3′ OH group. A capped dimer with a 3′ OH group will fit better into the closed active site of DcpS and will therefore be processed faster. The decapping rate that we measured for the capped trimer is independent of the 3′ end of the short mRNA fragment, as neither a capped timer with a 3′ OH group nor a capped trimer with a 3′ phosphate will fit into the closed active site of the enzyme (*SI Appendix*, Fig. S4). The biologically relevant drop in DcpS activity thus occurs between a capped dimer and a capped trimer, where we observe an over 100-fold reduction in turnover rates. The DcpS decapping rates of <0.3 per minute on long mRNAs is most likely too low to be of biological relevance.

Previous data by us and by others (10, 12, 13) have shown that DcpS needs to undergo a large conformational change to form a catalytically competent state. Based on methyl TROSY (19, 20) NMR data (Fig. 2), we here show that this state cannot be formed if the substrate has an mRNA body of three or more nucleotides. Structurally, this inability of DcpS to close around a long mRNA substrate is caused by steric clashes between the third base of the substrate and amino acids in both the N- and C-terminal domains of the enzyme. Steric clashes are a common mechanism in enzymes to ensure substrate selectivity, as has been described for e.g., the way lipases can accommodate lipids (29), the way DNA polymerase selects deoxyribonucleotides over ribonucleotides (30), or the way restriction enzymes discriminate between methylated and nonmethylated DNA (31). In addition, it is thought that mutations that change steric clashes in protein-ligand interfaces have played an important role in enzyme evolution (32). For DcpS, that mechanism is more advanced, as steric clashes do not interfere with the initial substrate recruitment event, but with the conformational changes that are required to remodel the enzyme into a catalytically active state. As a result, the enzyme can interact with long mRNA substrates but cannot process those efficiently. The narrow opening between the closed active site and the outside of the enzyme, that has previously been proposed as an exit tunnel is, however, too narrow to accommodate the mRNA body. Consequently, the activity of DcpS drops by two orders of magnitude when the length of the mRNA substrate exceeds two nucleotides. This steric gate in DcpS thus results in a precise mechanism that prevents longer mRNA substrates from being processed.

In summary, we show how a structural mechanism ensures that DcpS is only fully active on short mRNA fragments that are produced by the 3′ to 5′ mRNA degradation pathway and not mRNAs that are actively translated. Our data thus reveals that the enzymes that are involved in mRNA degradation pathway are mechanistically tightly coupled.

## Methods

### Protein Preparation.

BL21 (DE3) *Escherichia coli* cells were transformed with a plasmid that codes for a given DcpS variant (*SI Appendix*, Table S3) with a Tobacco etch virus (TEV) cleavable N-terminal His_6_-tag or His_6_-NusA-His_6_-tag. For activity assays and crystallization, cells were grown in lysogeny broth medium at 37 °C to OD_600_ = 0.6. Subsequently, protein expression was induced with 0.5 mM isopropyl β-d-1-thiogalactopyranoside and cells were shifted to 20 °C for 16 h. Cells were harvested by centrifugation and resuspended in buffer A (50 mM NaPO_4_, pH 8.0, 150 mM NaCl, 5 mM Imidazole) supplemented with 0.1% Triton X-100, 1 mM ethylenediaminetetraacetic acid, and lysozyme. After lysis by sonication, 2 mM MgSO_4_ was added, insoluble cell debris removed by centrifugation, and the supernatant applied to Ni-NTA resin that was equilibrated in buffer A. The resin was washed with buffer A, and the protein was eluted with buffer B (buffer A supplemented with 200 mM Imidazole). The eluted protein was supplemented with 0.5 mg of TEV protease and simultaneously dialyzed against 25 mM Tris pH 8.0, 75 mM NaCl, 1 mM dithiothreitol (DTT) at 4 °C. The cleaved affinity tag and TEV protease were removed by reverse Ni-affinity chromatography, after which the DcpS protein was subjected to size exclusion chromatography on a Superdex S200 column (GE Healthcare) in 25 mM Hepes pH 8.0, 25 mM NaCl.

The *S. cerevisiae* exo-9 complex was overexpressed from a single plasmid that carried the coding regions for all nine exosome core proteins (*SI Appendix*, Table S3), where the Rrp4 protein contains an N-terminal His_6_-TEV tag. The complete exo-9 complex was purified using Ni-NTA resin and size exclusion chromatography as described above for DcpS. The final exo-9 sample contained all nine proteins in equimolar amounts. Rrp44, the active subunits of the exosome complex, was overexpressed with an N-terminal His_6_-TEV tag and purified as described above for DcpS. The catalytically active exo-10 complex was prepared by mixing equimolar amounts of the exo-9 complex and Rrp44. The final exo-10 complex was obtained after an additional size-exclusion chromatography step in 20 mM Hepes pH 7.5, 200 mM NaCl, 1 mM DTT.

### RNA Preparation.

RNAs were prepared by in vitro transcription with T7 RNA polymerase and capped with vaccinia capping enzyme. In brief, equimolar amounts of an anti-sense strand primer that contains the T7 promoter sequence and a sense strand primer that contains the (reverse complementary) target RNA sequence and the T7 promoter (in reverse complement) were mixed at 1 µM concentration in 40 mM Tris pH 8, 5 mM DTT, 1 mM spermidine, 0.01% Triton X-100, 4 mM nucleoside triphosphates, 20–60 mM MgCl_2_, and 0.2 µM T7 RNA polymerase. Transcription reactions were incubated at 37 °C for 4 h, after which insoluble pyrophosphate was solubilized by addition of EDTA. The RNA product was precipitated at −20 °C in 0.3 M NaOAc pH 5.2 and 0.7 volumes of isopropanol or in 0.2 M NaCl and 3.5 volumes of EtOH (RNA below 10 nucleotides), followed by centrifugation at 9,000 × *g* for 1 h. The RNA pellet was washed with cold 75% ethanol, dried, and resuspended in water.

In vitro transcribed RNA was purified on a high-performance liquid chromatography (HPLC) system using anion exchange chromatography (22 × 250 mm DNAPac PA100 column; Dionex). The RNA was applied onto a heated column (80 °C) equilibrated in 20 mM Tris pH 8.0 and 5 M Urea and subsequently eluted with an NaCl gradient. The RNA-containing fractions were pooled, precipitated, desalted using a PD-10 column (GE Healthcare), and concentrated.

### Capping of the mRNA Body.

The purified RNA (*SI Appendix*, Table S4) was dissolved at a concentration of 20 μM in 50 mM Tris, pH 8, 5 mM KCl, 1 mM MgCl_2_, and 1 mM DTT, after which 0.5 mM GTP, 0.1 mM SAM, and capping enzyme were added (21). After incubation at 37 °C, the reaction was stopped and the enzyme removed by heating to 75 °C for 10 min and subsequently centrifuged for 10 min at 4,500 × *g*. The capped RNA was subsequently precipitated as described above and resuspended in an appropriate buffer.

### RNase A Cleavage.

Short capped RNAs were prepared by cleaving a longer capped RNA that contains, apart from a single U base, only GA bases. To that end, 50 ng of RNase A was added per nmol of RNA, followed by incubation at 37 °C for 10 min. Subsequently, the RNase A enzyme was removed by phenol-chloroform extraction and the 5′ and 3′ fragments of the RNA were separated by ion exchange chromatography as described above.

### DcpS Activity.

Decapping reactions were performed in 25 mM Hepes pH 8.0, 25 mM NaCl using a total volume of 110 μL. The total RNA concentration was between 20 and 200 µM, to which DcpS was added at a ratio of 1:200–1:10,000 depending on the decapping efficiency with the respective mRNA substrate. Reactions were incubated at 37 °C for 2 h and 20-µL samples for analysis were taken every 30 min. The decapping reaction was stopped by the addition of trifluoroacetic acid to a final concentration of 0.1% (vol/vol).

For reactions with mRNA substrates that contained an mRNA body of 1 or 2 nucleotides, the samples were analyzed with reverse-phase chromatography on a C18 column (Macherey Nagel). The column was equilibrated in 25 mM NH_4_Ac pH 5.3 at 40 °C, and a gradient from 0 to 60% acetonitril was used to separate the reaction products. For reactions with mRNA substrates that contained an mRNA body between 3 and 5 nucleotides, the samples were analyzed by anion exchange chromatography on a DNAPac PA200 RS (4.6 × 250 mm; Dionex). The column was equilibrated in 20 mM Tris pH 8.0 at 30 °C, and the reaction products were eluted based on a NaCl gradient from 0 to 0.5 M and 0.3–0.8 M for three and five nucleotides of RNA, respectively. For reactions with mRNA substrates that have an mRNA body over 10 nucleotides, the samples were analyzed on 20-cm-long 16% denaturing polyacrylamide gels. To visualize the RNA, the gels were stained with methyleneblue and quantified using GelBandFitter (www.uky.edu/∼kscamp3/gelbandfitter/Gelbandfitter_home.html) in Matlab. The reported enzyme activities are the average of at least three fully independent reaction series, with enzymes that were independently expressed and purified (*SI Appendix*, Table S1). This ensures that our decapping rates are highly accurate.

To compare the activity of *S. cerevisiae* WT DcpS with the Q130A and Q130A/N281A mutants, a capped 15-mer RNA with the sequence 5′-GGA​GAA​GAG​AAG​GAG-3′ was used (*SI Appendix*, Table S4). DcpS and RNA concentrations were 0.5 µM and 20 µM. The reaction was stopped by phenol/chloroform extraction and the RNA was analyzed by anion exchange chromatography as described above with the differences that a column temperature of 40 °C and a gradient from 420 to 560 mM NaCl over 25 min (flowrate 0.4 mL/min) were used.

### Coupled DcpS-Exosome Activity.

A capped 31-mer RNA (50 µM) was mixed with the eukaryotic exo-10 complex (0.5 µM) and/or DcpS (0.5 µM) in 20–40 µL of reaction buffer (20 mM Hepes pH 7.5, 200 mM NaCl, 1 mM DTT, 5 mM MgCl_2_). After 3 h at 37 °C, 10 µL of the reaction was injected into an HPLC system equipped with a DNAPac PA200 RS (4.6 × 250 mm; Dionex) column that was equilibrated in 20 mM Tris pH 8.0. The column was operated at 30 °C with a flow rate of 0.25 mL/min, and the RNA species were eluted using a NaCl gradient from 0 to 0.5 M. RNA was detected using the absorbance at 260 nm. AMP, GMP, and m^7^GMP standards were directly injected into the column.

### NMR Spectroscopy.

DcpS samples for NMR spectroscopy were prepared by overexpression of the catalytically inactive protein (H268N, H277N, or H258N for the *S. cerevisiae*, human, and *C. thermophilum* enzymes respectively; *SI Appendix*, Table S3) in D_2_O-based minimal medium that contained ^2^H^12^C glucose as the sole carbon source. Methyl groups were labeled by the addition of 60 mg/L α-ketobutyric acid (methyl ^13^CH_3_ labeled) and 100 mg/L methionine (^1^H, methyl-^13^C labeled) 1 h prior to induction. The expression and purification of these proteins was performed as described above. NMR samples contained between 50 and 500 µM protein in 100% D_2_O, 25 mM Hepes pH 8.0, and 25 mM NaCl. Methyl TROSY NMR spectra (19) were recorded on Bruker Avance 600 and 800 MHz spectrometers at 20 °C or 25 °C. Typical NMR spectra were recorded with a total carbon chemical shift evolution time of 25 ms. NMR data were processed using the NMRpipe program-suit (33).

### Protein Crystallization.

The 1:1 complex of DcpS from *S. cerevisiae* (residues 8–350; H268N; *SI Appendix*, Table S3) with m^7^GpppGU RNA (*SI Appendix*, Table S4) was crystallized at a concentration of 5 mg/mL in 10 mM Hepes pH 7.5, 100 mM NaCl, 1.6 M NH_4_SO_4_. DcpS from *C. thermophilum* was crystallized at a concentration of 13 mg/mL in 100 mM BisTRIS pH 5.5, 200 mM NH_4_Ac, 25% PEG 3350. Crystals were flash frozen in 30% glycerol, and diffraction data were collected at the beamline PXII of the Swiss Light Source at a wavelength of 1 Å at 100 K. Data were processed using XDS (34), and the structures were determined by molecular replacement using Phaser (23). For the structure of DcpS in complex with m^7^GpppGU, the structure with Protein Data Bank (PDB) ID code 5BV3 (10) was used as a search model. For the structure of the *C. thermophilum* enzyme, the structure with PDB ID code 1XML (13) was used. Iterative model building was performed using the program Coot (35), and the structure refinement was performed using the programs Refmac (36) and Phenix (37). All figures showing structural data were prepared using Pymol (https://pymol.org/2/).

## Supplementary Material

Supplementary File

## Data Availability

Atomic coordinates and structure factors have been deposited in the PDB under accession codes 6GBS (Apo-DcpS from *C. thermophilum*) and 6TRQ (DcpS: m^7^GpppGU complex from *S. cerevisiae*).
