# Supplementary material for: Molecular basis of the selective processing of short mRNA substrates by the DcpS mRNA decapping enzyme
Source: Proc Natl Acad Sci U S A. 2020 Jul 28;117(32):19237–44. doi: 10.1073/pnas.2009362117 (PMC7431086; doi:10.1073/pnas.2009362117)
Supplement: Supplementary File [file pnas.2009362117.sapp.pdf]

Supporting information for

Molecular basis of the selective processing of short mRNA substrates by the DcpS decapping enzyme.

Authors:

Anna-Lisa Fuchs[1,3], Jan Philip Wurm[1], Ancilla Neu[2,4], Remco Sprangers[1,*]

Author Affiliations:

[1] Department of Biophysics I, Regensburg Center for Biochemietry, University of Regensburg, 93053 Regensburg, Germany. [2] Max Planck Institute for Developmental Biology, Spemannstrasse 35, 72076 Tübingen, Germany. [3] Current address: TU Munich, Department of Informatics, Boltzmannstr. 3, Garching, Germany. [4] Current address: Rentschler Biopharma SE, Laupheim, Germany.

Corresponding Author:

Remco Sprangers, E-mail: remco.sprangers@ur.de, Phone: +49 (0)941 943 7751.

This pdf file includes:

Tables

S1: mRNA decapping rates

S2: Data collection and refinement statistics.

S3: Plasmids used or protein expression

S4: Primers/ Plasmids used for RNA production

Figures

S1: Fitting of mRNA decapping rates

S2: Interaction between DcpS and a capped trimer

S3: Electron density around the capped dimer

S4: Model of a longer capped mRNA in the active site of DcpS

S5: Activity of DcpS tunnel mutants

S6: Sequence alignment

S7: The products of the exosome are substrates for DcpS.

www.pnas.org/cgi/doi/10.1073/pnas.2009362117

Table S1. mRNA decapping rates

Length of the RNA body	Turnover rate (min^{-1})	RNA detection method	# measurements
1	30.82 (5.83)	HPLC RP (C18)	5
2	7.73 (2.90)	HPLC RP (C18)	3
3	0.20 (0.12)	HPLC IEX (Dionex)	3
5	0.03 (0.01)	HPLC IEX (Dionex)	3
15	0.37 (0.13)	UREA PAGE	5
30	0.26 (0.15)	UREA PAGE	3
31	0.33 (0.04)	UREA PAGE	3
Average of 3 to 31	0.26 (0.17)	Mixed	17

In vitro turnover rates for DcpS mediated mRNA decapping were determined for substrates with an RNA body between 1 and 31 nucleotides (see methods for the preparation of the substrates). For each measurement, mRNA substrate and *S. cerevisiae* DcpS enzyme (both highly purified) were mixed and the reaction progression was assessed at 5 different time-points and the turnover rates were extracted. It was ensured that the reaction kinetics were in the linear regime. For each RNA, 3 to 5 independent measurements with different enzyme batches were performed to extract the error in the turnover rates. The numbers in parenthesis represent the standard deviation.

Table S2. Data collection and refinement statistics.

	S.c. DcpS:m^{7}GpppGU complex (6TRQ)	C.t. Dcps (6GB6)
**Data collection**		
Wavelength (Å)	1.00	1.00
Resolution range (Å)	47.31 - 2.944 (3.049 - 2.944)	46.3 - 1.946 (2.016 - 1.946)
Space group	P 21 21 21	P 1
Unit cell: a, b, c (Å)<br>α,β,γ(°)	87.761 104.103 189.223<br>90 90 90	52.595 69.719 70.947<br>104.484 101.391 111.611
Total reflections	499207 (49256)	208086 (20935)
Unique reflections	37383 (3591)	60704 (5929)
Multiplicity	13.4 (13.7)	3.4 (3.5)
Completeness (%)	99.54 (97.29)	96.56 (94.12)
Mean I/sigma(I)	9.12 (1.23)	9.80 (2.19)
Wilson B-factor	66.78	28.97
R-merge	0.3122 (2.397)	0.08574 (0.6772)
R-meas	0.3245 (2.488)	0.1024 (0.802)
R-pim	0.08787 (0.6632)	0.05501 (0.424)
CC1/2	0.995 (0.693)	0.996 (0.761)
CC*	0.999 (0.905)	0.999 (0.93)
**Refinement**		
Reflections used in refinement	37324 (3585)	60833 (5926)
Reflections used for R-free	1870 (179)	3043 (296)
R-work	0.2043 (0.3565)	0.1827 (0.2931)
R-free	0.2633 (0.3946)	0.2165 (0.3173)
CC(work)	0.953 (0.806)	0.968 (0.861)
CC(free)	0.920 (0.809)	0.959 (0.750)
Number of non-hydrogen atoms	10917	5714
macromolecules	10905	5402
ligands	10	55
solvent	2	257
Protein residues	1309	663
RMS(bonds)	0.008	0.007
RMS(angles)	1.31	0.87
Ramachandran favored (%)	95.17	98.02
Ramachandran allowed (%)	3.82	1.98
Ramachandran outliers (%)	1.01	0.00
Rotamer outliers (%)	3.01	1.06
Clashscore	14.55	4.32
Average B-factor	82.52	34.15
macromolecules	82.50	33.87
ligands	112.92	57.09
solvent	37.80	34.95

Statistics for the highest-resolution shell are shown in parentheses. One crystal was used for the determination of each structure. The table was generated using the table_one utility as implemented in phenix (1).

Table S3. Plasmids used for protein expression.

Protein	Organism	Uniprot ID	Purification Tags	Mutations	Used in	#
DcpS	*Saccharomyces cerevisiae*	Q06151	N-His$_6$-NusA-His$_6$-TEV	-	Fig. 1B, 1C, 1D, 5	100
DcpS	*Saccharomyces cerevisiae*	Q06151	N-His$_6$-NusA-His$_6$-TEV	H268N	Fig. 2A, 2B	109
DcpS	*Saccharomyces cerevisiae*	Q06151	N-His$_6$-NusA-His$_6$-TEV	Q130A	Fig. 3D	2051
DcpS	*Saccharomyces cerevisiae*	Q06151	N-His$_6$-NusA-His$_6$-TEV	Q130A, N281A	Fig. 3D	2178
DcpS	*Saccharomyces cerevisiae*	Q06151	N-His$_6$-NusA-His$_6$-TEV	H268N Δ1-7	Fig. 3A	416
DcpS	*Homo sapiens*	Q96C86	N-His$_6$-NusA-His$_6$-TEV	H277N	Fig. 4D	1542
DcpS	*Chaetomium thermophilum*	G0S8A3	N-His$_6$-TEV	H258N	Fig. 4A, 4B, 4C	1670
Exo-9	*Saccharomyces cerevisiae*	P38792 (Rrp4)<br>Q08285 (Rrp40)<br>P53859 (Csl4)<br>P46948 (Rrp41)<br>Q12277 (Rrp42)<br>P25359 (Rrp43)<br>Q05636 (Rrp45)<br>P53256 (Rrp46)<br>P48240 (Mtr3)	N-His$_6$-TEV (Rrp4)	-	Fig. 5	660
Rrp44	*Saccharomyces cerevisiae*	Q08162	N-His$_6$-SUMO-TEV	-	Fig. 5	446
Capping complex (2)	*Vaccinia virus*	P04298 (D1)<br>P04318 (D12)	N-His$_6$-TEV (D1)	-	Methods	1857

TEV: Tobacco Etch Virus protease site (ENLYFQ|G, where the "|" is the protease cleavage site). #: internal reference.

Table S4. Primers/ plasmids used for RNA production

<table>
<tr><th>RNA</th><th>Primer sequence (anti sense)</th><th>RNA sequence</th><th>Fig.</th><th>#</th></tr>
<tr><td>2-mer</td><td>cttctttcttcccttccttcctctccttac<u>tatagtgagtcgtattacg</u></td><td>gu|aaggagaggaaggaagggaagaaaga</td><td>1, 2, 3, 4</td><td>1619</td></tr>
<tr><td>3-mer</td><td>cttctttcttcccttccttcctctcctacc<u>tatagtgagtcgtattacg</u></td><td>ggu|aggagaggaaggaagggaagaaagaag</td><td>1, 3, 4</td><td>7</td></tr>
<tr><td>5-mer</td><td>cttctttcttcccttccttcctctcactcc<u>tatagtgagtcgtattacg</u></td><td>ggagu|gagaggaaggaagggaagaaagaag</td><td>1</td><td>8</td></tr>
<tr><td>10-mer</td><td>cttctttcttcccttccttcactctcctcc<u>tatagtgagtcgtattacg</u></td><td>ggaggagagu|gaaggaagggaagaaagaag</td><td>1</td><td>9</td></tr>
<tr><td>15-mer</td><td>cttctttcttcccttaccttcctctcctcc<u>tatagtgagtcgtattacg</u></td><td>ggaggagaggaaggu|aagggaagaaagaag</td><td>1</td><td>10</td></tr>
<tr><td>15-mer</td><td>ctccttctcttctcc<u>tatagtgagtcgtatta</u></td><td>ggagaagagaaggag</td><td>3</td><td>4</td></tr>
<tr><td>30-mer</td><td>cttctttcttacccttccttcctctcctcc<u>tatagtgagtcgtattacg</u></td><td>ggaggagaggaaggaagggtaagaaagaag</td><td>1</td><td>123</td></tr>
<tr><td>31-mer</td><td>Transcribed from plasmid</td><td>gaaggagaggaaggaaagggaagaaagaagg*</td><td>1, 5</td><td>33</td></tr>
</table>

The verticale bar "|" in the RNA sequence indicates that the RNA is cleaved at that position with RNase A. The T7 promoter sequence is underlined. * refers to a cyclic phosphate at the 3' end of the RNA that is due to the cleavage of the precursor RNA 3' with a ribozyme. #: internal reference.

Capped 1-mer

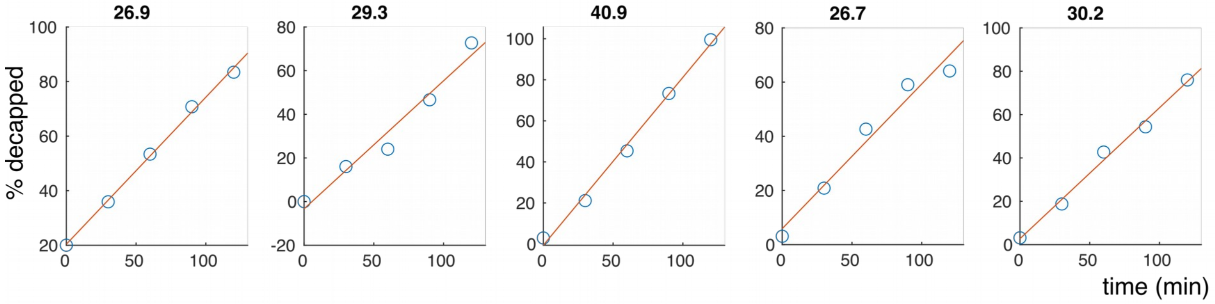

Capped 2-mer

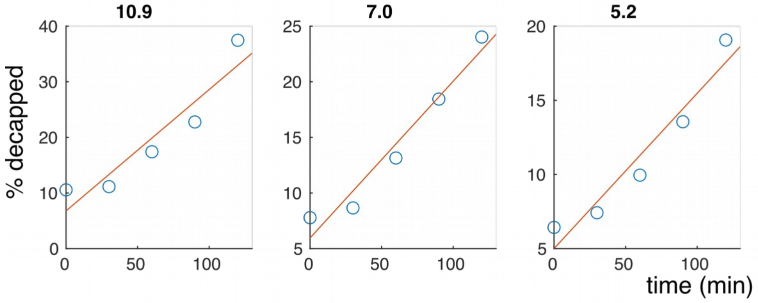

Capped 3-mer

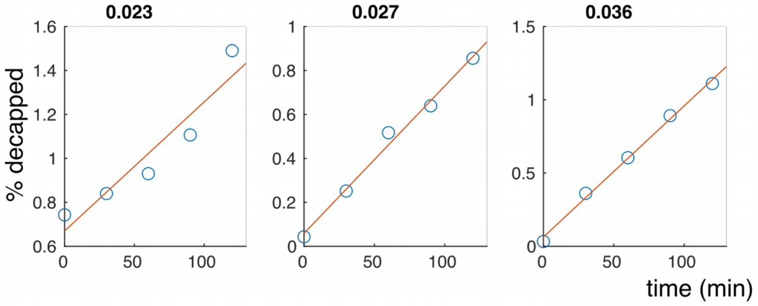

Capped 5-mer

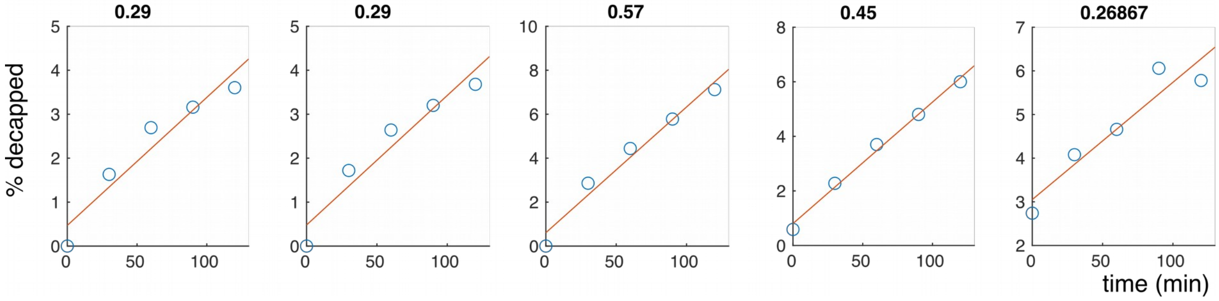

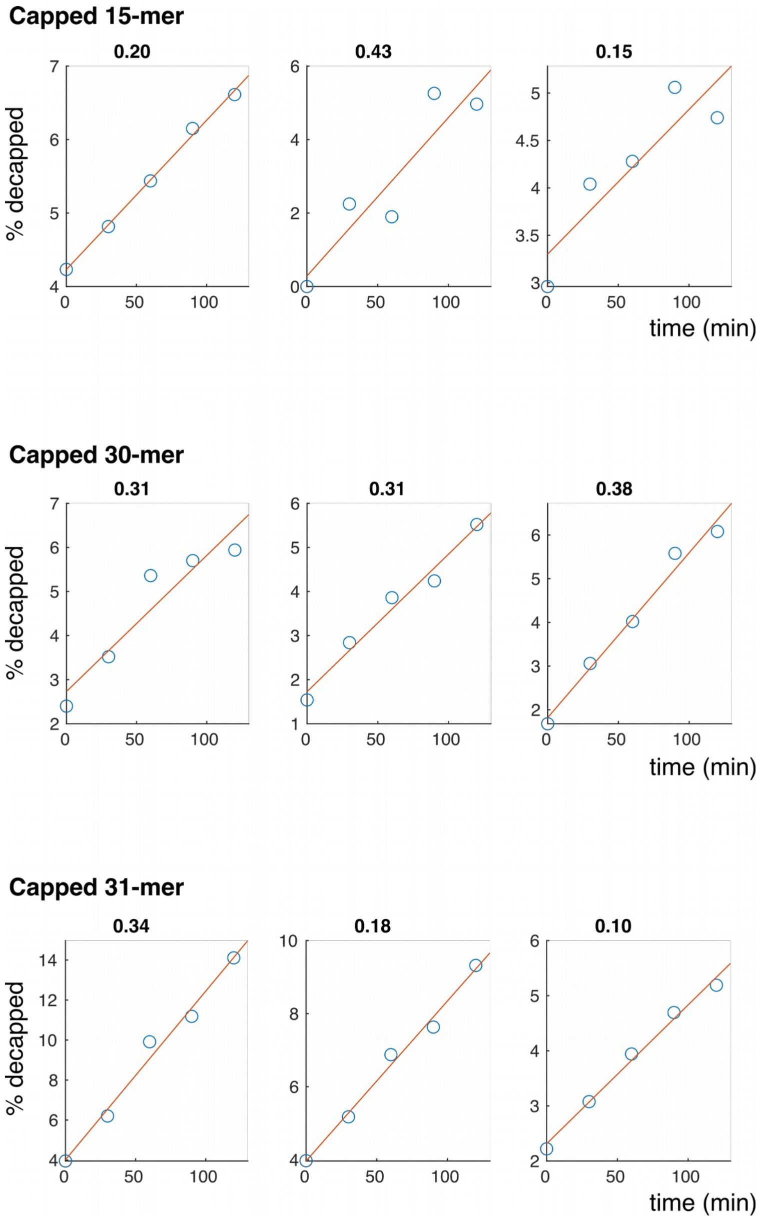

Fig. S1. Fitting of mRNA decapping rates.

Experimental data that was used to determine the DcpS decapping rates (*S. cerevisiae*) for the different substrates. The fitted degradation rates (in min^{-1}) are indicated on top of the graphs. The rates reported in Fig. 1 are the average (standard deviation) of the individual rates that have been shown here. The experimental details can be found in the Methods section and Table S1. All repeat experiments have been performed by pipetting and analyzing the reactions independently. In all single experiments enzymes have been independently purified.

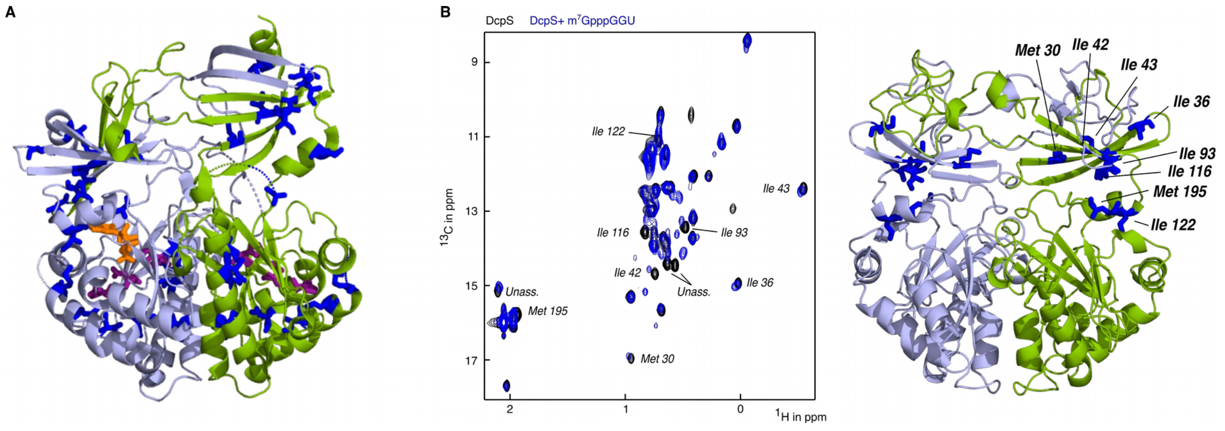

Fig. S2. Interaction between DcpS and a capped monomer/ capped trimer.

(A) Structure of the asymmetric form of DcpS (3). Residues (isoleucine and methionine) that experience chemical shift perturbations upon the interaction of DcpS with a capped monomer are shown as blue sticks. The m7GDP ligand in the active site is shown in orange. The active site residues are shown in purple.

(B) Left: Methyl TROSY (4, 5) NMR spectrum of the *S. cerevisiae* DcpS enzyme in the absence (black) and presence (blue) of a capped tri-nucleotide. Assignments for residues that undergo chemical shift perturbations are indicated. Unass: a resonance for which a resonance assignment is not available.

Right: Residues that sense the presence of the capped tri-nucleotide plotted on a model of the symmetric form of the S.c. DcpS enzyme. The substrate binds at the cleft between the N- and C- terminal domains, but does not induce the formation of a closed active site. The model of the symmetric apo-structure of the yeast DcpS enzyme was generated using Modeller (6) with the apo-structure of the human enzyme (7) (PDBID: 1XML) as template.

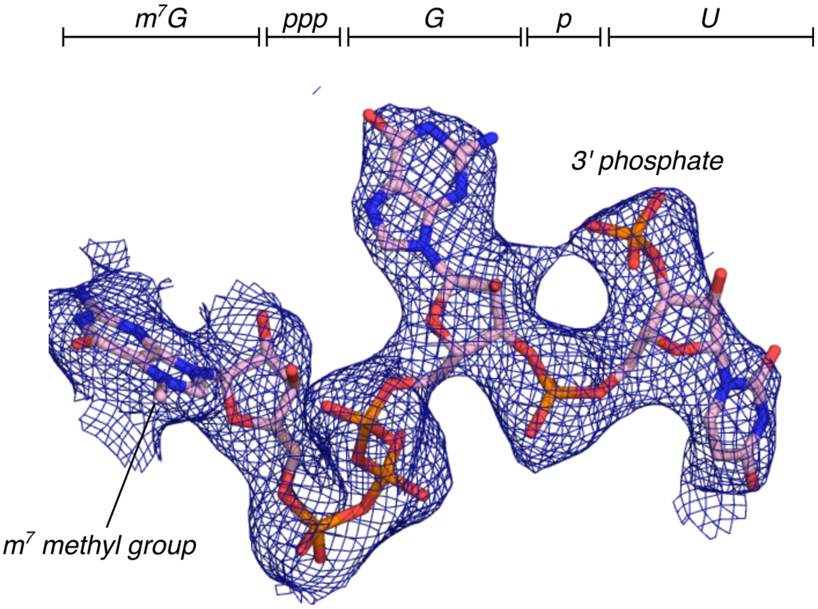

Fig. S3. Electron density around the capped dimer.

$2F_O$-F_C electron density around the m[7]GpppGU substrate in the closed and catalytically competent active site, contoured at 1σ. Note that the substrate that was used in the crystallization and activity studies here contains a 3' phosphate group. The products that are produced by the exosome complex contain a 3' OH group.

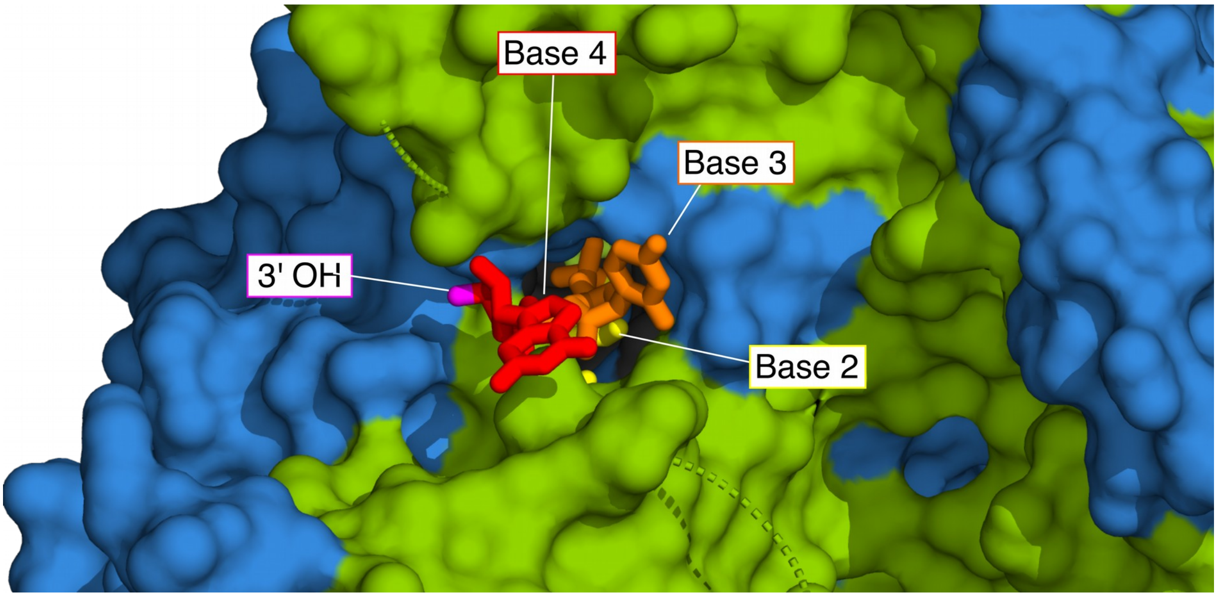

Fig. S4. Model of a longer capped mRNA in the active site of DcpS.
A capped 4-mer RNA is docked into the structure of the complex of the *S. cerevisiae* DcpS enzyme and capped 2-mer. The m^{7}cap and the first base are not visible, as these reside too deep inside the closed active side. Bases 1 and 2 (yellow) reside fully inside the DcpS cavity. Base 3 (orange) occupies the narrow exit channel and experiences a large number of steric clashes with side- and main-chain atoms in DcpS. Base 4 (red) is fully outside the exit channel and has a freely accessible 3' OH group, such that the remaining of the RNA body does not come in close spatial proximity to DcpS. The RNA was modeled onto the structure of DcpS enzyme in complex with m^{7}GpppGU using CYANA. To that end, the first base of a 4-mer RNA was superimposed onto the first base of the capped 2-mer. Subsequently the "regularize" script in CYANA was used to place bases 2 to 4 into the closed conformation of the complex (8).

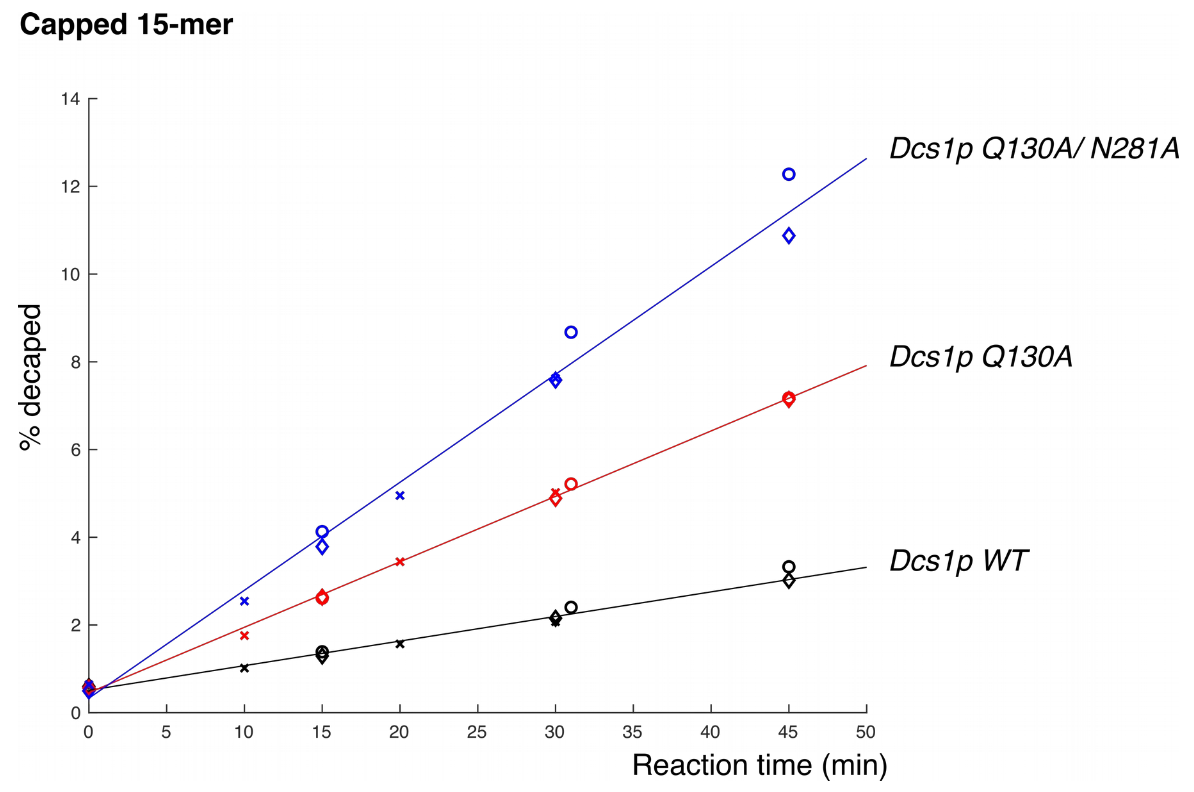

Fig. S5. Activity of DcpS tunnel mutants.

Activity data from the DcpS tunnel mutants. The WT enzyme is shown in black (decapping rate: 0.11 (0.02) min^{-1}; in agreement with the experiments based on UREA-PAGE analysis, Fig 1 and Table S1), the single Q130A mutant in red (decapping rate: 0.30 (0.01) min^{-1}) and the double Q130A/ N281A mutant in blue (decapping rate 0.49 (0.05) min^{-1}). The different symbols (circle, cross, diamond) indicate the three independent measurements to determine the decapping rates. The drawn line is the best fit to all measurements. The fractions of the capped and decapped RNA have been determined using an HPLC analysis (see methods).

For completeness, we would like to mention that we also assayed the activity of the S31A, Q129A/F132A, K126A/N281A, A130A/F132A, K126A/P227G, A129A/Q130A, S31A/N281A, Q130A/K287A, S31A/K275A, K126A/Q129A and N281A/K287A DcpS mutants, however, for none of those mutants we found a significant increase in the DcpS mediated decapping rates. Reduced decapping rates can, in part, be due to reduced enzyme stability, especially in the case of double mutants. As an example: the N281A/K287A DcpS enzyme appeared unfolded. We have not accurately determined degradation rates for those mutants as they were not of prime interest to us.

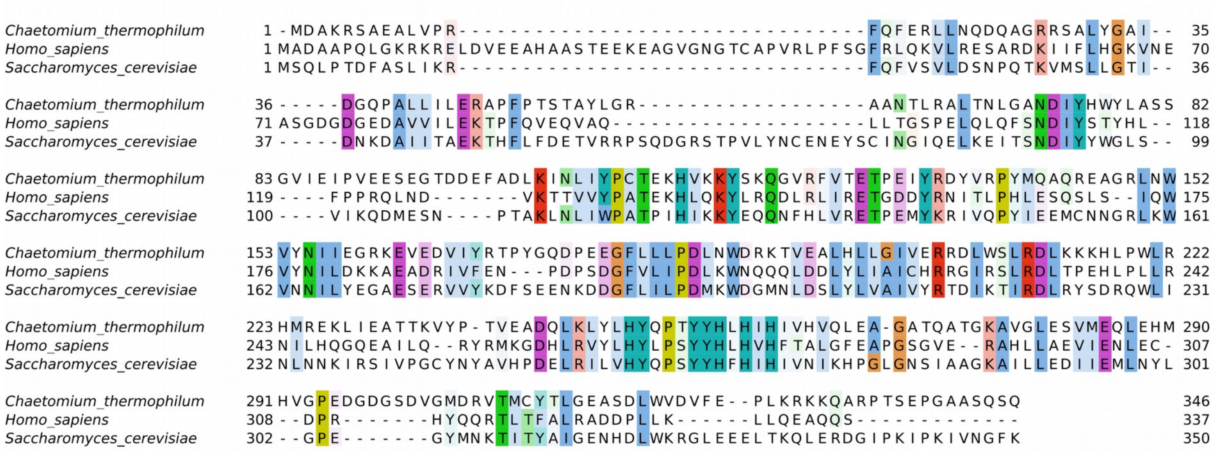

Fig. S6. Sequence alignment.

Alignment of the DcpS sequences from *S. cerevisiae*, *C. thermophilum* and *H. sapiens*. The intensity of the colors corresponds to the level of sequence conservation. The alignment was prepared using Jalview (9). The pairwise identity between the *S. cerevisiae*, and *C. thermophilum* enzymes is 32%, between the *S. cerevisiae* and human proteins 29% and between the *C. thermophilum* and the human protein 29%.

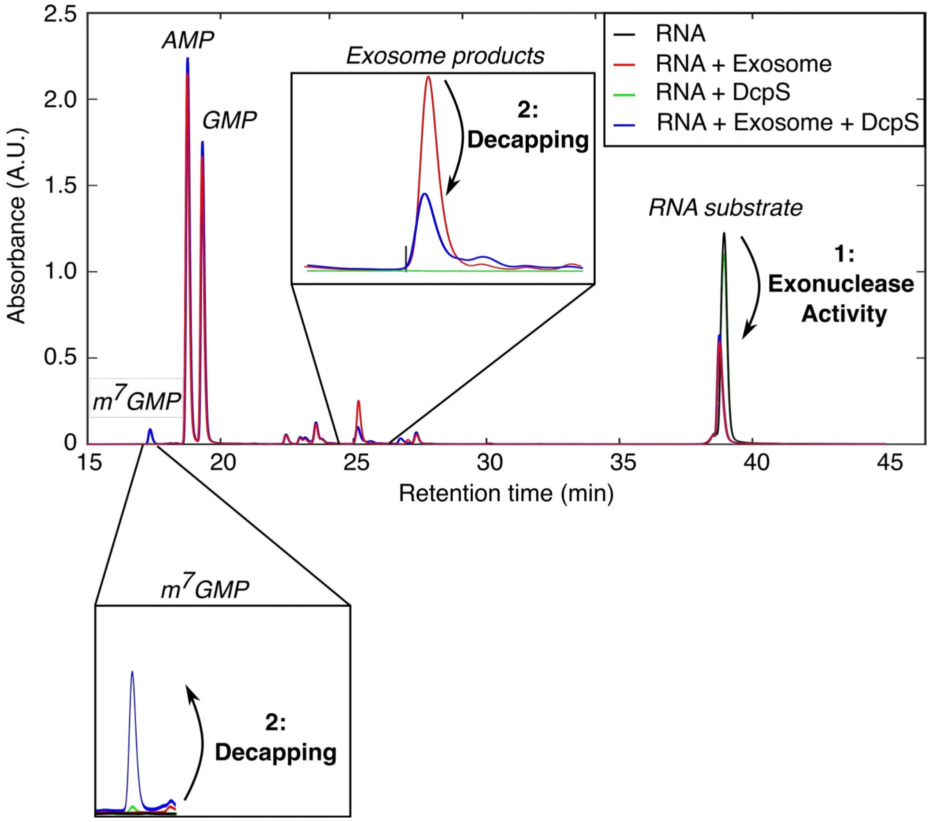

Fig. S7. The products of the exosome are substrates for DcpS.

Reaction mixtures of an mRNA with a body of 31 bases are analyzed by ion exchange chromatography on an HPLC. The mRNA was incubated with or without the cytoplasmic exosome (exo-10) complex and with or without DcpS. The mRNA alone (black) elutes around 38 min. Addition of the exosome to the mRNA (red) results in a reduction of the mRNA (1: exonuclease activity) and the appearance of shorter fragments (exosome products). In the presence of DcpS (blue), these exosome products are decapped (2: Decapping), showing that DcpS is active on the product from the exosome. Note the appearance of a peak for the m^7GMP decapping product. The mRNA is not decapped at significant rates in the absence of the exosome (green), which is in agreement with the inability of DcpS to process long mRNA substrates (note the very small m^7GMP peak, that results from the residual activity of DcpS on long mRNAs). Note that the exosome reaction has been terminated before the complete mRNA substrate was degraded. This was done to prevent that the released products of the exosome could be further processed by the exosome in subsequent degradation rounds.

Supporting references

1. P. D. Adams, *et al.*, PHENIX: A comprehensive Python-based system for macromolecular structure solution. *Acta Crystallographica Section D: Biological Crystallography* **66**, 213–221 (2010).

2. A.-L. Fuchs, A. Neu, R. Sprangers, A general method for rapid and cost-efficient large-scale production of 5' capped RNA. *RNA* **22**, 1454–1466 (2016).

3. A. Neu, U. Neu, A.-L. Fuchs, B. Schlager, R. Sprangers, An excess of catalytically required motions inhibits the scavenger decapping enzyme. *Nat. Chem. Biol.* **11**, 697–704 (2015).

4. V. Tugarinov, P. M. Hwang, J. E. Ollerenshaw, L. E. Kay, Cross-Correlated Relaxation Enhanced ^{1}H−^{13}C NMR Spectroscopy of Methyl Groups in Very High Molecular Weight Proteins and Protein Complexes. *J. Am. Chem. Soc.* **125**, 10420–10428 (2003).

5. S. Schütz, R. Sprangers, Methyl TROSY spectroscopy: A versatile NMR approach to study challenging biological systems. *Progress in Nuclear Magnetic Resonance Spectroscopy*, S0079656519300470 (2019).

6. A. Sali, T. L. Blundell, Comparative protein modelling by satisfaction of spatial restraints. *J. Mol. Biol.* **234**, 779–815 (1993).

7. N. Chen, M. a. Walsh, Y. Liu, R. Parker, H. Song, Crystal structures of human DcpS in ligand-free and m7GDP-bound forms suggest a dynamic mechanism for scavenger mRNA decapping. *Journal of Molecular Biology* **347**, 707–718 (2005).

8. P. Güntert, L. Buchner, Combined automated NOE assignment and structure calculation with CYANA. *Journal of Biomolecular NMR* **62**, 453–471 (2015).

9. A. M. Waterhouse, J. B. Procter, D. M. A. Martin, M. Clamp, G. J. Barton, Jalview Version 2--a multiple sequence alignment editor and analysis workbench. *Bioinformatics* **25**, 1189–1191 (2009).
